# Seroprevalence and risk factors for *Neospora caninum* infection in dogs in rural northeastern mainland China

**DOI:** 10.1051/parasite/2019034

**Published:** 2019-05-30

**Authors:** Xiang Gao, Hongbin Wang

**Affiliations:** Department of Veterinary Surgery, Northeast Agricultural University Harbin 150030 Heilongjiang PR China

**Keywords:** Dogs, *Neospora caninum*, Logistic models, Risk factors, Prevention and control

## Abstract

Although *Neospora caninum* is an important veterinary pathogen, veterinarians in various areas including in Mainland China lack a full understanding of neosporosis distribution in dog populations. This study aims to determine the emergence of anti-*N. caninum* antibodies in canine populations classified based on breeders, herdsmen, and huntsmen in northeast mainland China. In addition, the risk factors associated with seropositivity were explored. An indirect immunofluorescent antibody test (IFAT) was performed on canine serum to determine seroprevalence. Logistic regression models were used to collect and analyze individual and management data, in order to determine high-reliability predictors of seroprevalence as well as the level of anti-*N. caninum* antibodies. Among the 476 dogs tested, 95 (20%) were seropositive. Mixed breed (OR 1.53), former strays (OR 1.38), dogs living on cattle farms (OR 2.30), hunting dogs (OR 1.22) as well as raw meat feeding (OR 1.66) were correlated (*p* < 0.05) with *N. caninum* infection. Interestingly, the seropositivity of dogs on cattle farms was higher (28%) than that of those (24.8%) living in breeding facilities (*p* < 0.05). A large number of seropositive dogs were found on cattle farms in the study region, suggesting horizontal transmission between dogs and cattle. Therefore, this source of infection should be studied further, and should be a strong consideration in differential diagnoses of dogs raised on cattle farms.

## Abstract (fr)

Bien que *Neospora caninum* soit un agent pathogène vétérinaire important, les vétérinaires de diverses régions, dont la Chine continentale, ne comprennent pas parfaitement la répartition de la néosporose dans les populations de chiens. Cette étude vise à déterminer l’émergence d’anticorps anti-*N. caninum* dans les populations canines classées comme reproducteurs, bergers et chasseurs du nord-est de la Chine. De plus, les facteurs de risque associés à la séropositivité ont été explorés. Un test d’immunofluorescence indirecte (IFAT) a été réalisé sur les sérums des chiens afin de déterminer la séroprévalence. Des modèles de régression logistique ont été utilisés pour collecter et analyser des données individuelles et de gestion, afin de déterminer les prédicteurs de fiabilité élevée de la séroprévalence ainsi que le niveau d’anticorps anti-*N. caninum*. Parmi les 476 chiens testés, 95 (20 %) étaient séropositifs. La race mélangée (OR 1,53), les animaux auparavant errants (OR 1,38), vivant dans des fermes d’élevage bovin (OR 2,30), les chiens de chasse (OR 1,22) ainsi que l’alimentation en viande crue (OR 1,66) étaient corrélés (*p* < 0,05) avec l’infection à *N. caninum*. Fait intéressant, la séropositivité des chiens dans les fermes d’élevage bovin était supérieure (28 %) à celle de ceux (24,8 %) vivant dans des élevages de chiens (*p* < 0,05). Un grand nombre de chiens séropositifs ont été trouvés dans des fermes d’élevage bovin dans la région étudiée, suggérant une transmission horizontale entre chiens et bovins. Par conséquent, cette source d’infection doit être étudiée plus avant et constituer un facteur important dans le diagnostic différentiel des chiens élevés dans des fermes d’élevage bovin.

## Introduction

*Neospora caninum* is a protozoan cyst-forming apicomplexan parasite. Colonization by the parasite results in neosporosis, which is a clinically important disease in cattle (*Bos taurus*) [1, 6]. Neosporosis is considered to be a critical infectious cause of abortion in cattle worldwide. The major losses resulting from neosporosis are associated with reproductive failure [7]. Numerous other indirect costs associated with neosporosis include replacement cost, re-breeding and reduced milk yield (if the animals are culled), as well as a reduction in the value of the infected animals [25–27]. A recent study has estimated that the total median economic influence of *N. caninum* in both the beef and dairy industries is approximately US $1.298 billion per annum. The dairy industry has incurred the most significant annual losses of approximately US $842 million, whereas the beef industry has sustained annual losses of approximately US $455 million [23].

Oocysts are the key factor in the epidemiology of neosporosis, generated by sexual reproduction in dog (*Canis lupus familiaris*), Gray wolf (*Canis lupus*), Australian dingo (*Canis lupus dingo*) and Coyote (*Canis latrans*) intestinal epithelial cells. Each of these species have been confirmed as hosts of *N. caninum* [8, 12, 15]. Oocysts are shed in an un-sporulated form. However, they can sporulate outside the host within 24 h [20]. It has been estimated that an infected dog can produce as many as 5 × 10^5^ oocysts after eating tissue from an infected intermediate host. Only asexual life stages of *N. caninum* have been observed in intermediate hosts (tachyzoites and encysted bradyzoites), including cattle [5, 31]. Previous studies have shown that the prevalence of *N. caninum* in dogs ranges anywhere from 2.1 to 36.4% in Europe, and 0.54 to 34.91% in central China [2, 10, 11, 17, 22, 28–30].

Dogs are commonly kept as companion animals throughout the world, or employed as working animals to assist farmers, herdsmen, and hunters. To prevent infection of dogs and thereby reduce oocyst shedding by dogs, the risk factors for infection need to be well understood. In the current study, the risk factors affecting *N. caninum* infection of dogs in rural Heilongjiang province were assessed. This region lies in the northeast of mainland China, and is one of the largest beef and dairy production regions in the country. The findings of the risk factor analysis will provide the necessary information to develop effective surveillance and control strategies for the control of neosporosis.

## Materials and methods

### Ethics statement

All the experimental procedures in animals were conducted following the Ethical Principles in Animal Research issued by Northeast Agricultural University.

### Study population

The study included samples collected from 32 villages throughout Heilongjiang between January and August 2018. All dogs at participating clinics were qualified to participate in the study. Permission was obtained from the dog owners prior to collection of samples, and a medical history questionnaire was completed for each subject. A total of 476 blood samples were collected from domestic dogs in the study area. Blood samples were then centrifuged, and sera were collected and stored at −80 °C for future analysis.

### Serologic testing

An indirect immunofluorescent antibody test (IFAT, FULLER Laboratories, California, USA) was used to detect serum antibodies against *N. caninum*. Dog sera showing a fluorescent signal at a titer of 1:50 were considered to be seropositive [24, 32]. The IFAT analysis was conducted according to the manufacturer’s instructions. Briefly, the slides were incubated with the serum samples for 30 min at 37 °C. Next, the FITC anti-dog conjugate was added to each well. The samples were then incubated for 30 min at 37 °C, and were then washed three times with phosphate-buffered saline (PBS), and mounting medium and coverslips were applied. Positive reactions were determined using UV light microscopy at 400× magnification to read these samples under a fluorescence microscope. The test exhibited sensitivity of 98%, and specificity of 99% [24]. Analyses of all sera were performed in duplicate.

### Questionnaires

A self-administered questionnaire was provided to dog owners to aid in the identification of risk factors associated with *N. caninum* infection. The questionnaire addressed topics such as housing (environment type, whether there are other dogs in the household or around the house), contact with young dogs, outdoor access, hunting behavior, raw meat feeding, if the pet was formerly a stray, and the availability of a litter tray. Gender, breed, spay/neuter history, and vaccination status data were also collected.

### Data analyses

For the majority of the variables examined, considering the similar seroprevalence and small sample sizes for each category, the client responses on the questionnaires were converted to categorical data. For example, outdoor access was divided into two categories, “no” responses corresponded to indoor dogs and dogs with limited outdoor access (like a balcony, and areas allowing no contact with other dogs), and “yes” only refers to dogs with free outdoor access. The vaccination status was limited to the category of “yes” which included annual vaccination and one-off vaccination, whereas “no” consisted of unvaccinated animals. The use of litter tray responses were categorized as seldom or never use into “no,” and always or often use into “yes”. The number of dogs surrounding a household was categorized as “no” (none), and “yes” (one or more). In an owner fed their dog(s) raw meat at all during the year, this was considered a “yes” response, and no raw meat feeding was a “no.” For hunting, “yes” involved dogs that bring prey home or eat prey animals. Missing values in the analysis were coded using an indicator variable.

To identify predictors of seropositivity for dogs infected with *N. caninum*, bivariable logistic regression analyses were conducted. All variables reaching a significance level of *p* ≤ 0.20 (measured by the likelihood-ratio test, LRT) were selected for incorporation into the multivariable logistic regression model. The model was reduced by backwards elimination of non-significant variables (*p* > 0.05 calculated by LRT). Hosmer and Lemeshow goodness of fit tests were used to assess the model fit. Logistic regression analysis was conducted using SPSS 18.0 (PASW Statistics, Chicago, IL, USA).

## Results

Among the 476 dogs included in the study, 95 (20%) were seropositive for *N. caninum* antibodies. Serum samples with a titer of at least 1:50 were considered to be positive. In 7 of the 32 total villages analyzed, no *N. caninum* antibodies were detected. In the remaining 25 villages, seroprevalence ranged from 3.7 to 32.1%.

According to the bivariate analyses, breed, former stray dogs, dogs living on cattle farms, raw meat feeding, outdoor access, hunting, hours outside each day, litter tray use, presence of one dog or more dogs in the household, and contact with young dogs were observed to exert a significant effect on seroprevalence (*p* < 0.20) (Table 1). In Table 2, the final model built on multivariate logistic regression analysis (Hosmer and Lemeshow goodness of fit test *p* = 0.674) is presented. Significant predictors of seropositivity included breed, former stray dog, dogs living on cattle farms, hunting behavior, and raw meat feeding (*p* < 0.05) (Table 2).

**Table 1:** Response rate per variable, percentage of dog serum samples positive for antibodies against *Neospora caninum* (prevalence) by variable category, age-adjusted odds-ratios (OR) with 95% confidence interval, and *p*-values (based on likelihood ratio tests) for those variables in the bivariable logistic regression analysis.

Variable	Prevalence (%)	OR (95% CI)	*p-*value
Age			>0.20
<3 years	16.4	Reference	
>3 years	20.1	1.37 (1.00-1.89)	
Missing	19.8	1.22 (0.74-1.98)	
Sex			>0.20
Male	18.6	Reference	
Female	32.2	2.64 (0.86–8.12)	
Missing	21.3	1.24 (0.91–1.70)	
Former stray			<0.20
No	10.7	Reference	
Yes	35.4	4.45 (1.75–11.3)	
Missing	19.9	2.07 (1.12–3.83)	
Dogs living on cattle farms			<0.20
No	14.1	Reference	
Yes	27.9	1.90 (1.07–3.37)	
Missing	17.5	1.18 (0.28–5.03)	
Dogs in the household			<0.20
No	25.9	Reference	
1 or 2	13.8	0.77 (0.34–1.71)	
3 or more	9.2	0.32 (0.17–0.63)	
Missing	19.1	0.89 (0.39–2.01)	
Raw meat			<0.20
No	10.2	Reference	
Yes	21.6	2.54 (0.73-8.86)	
Missing	20.5	2.36 (0.48-11.5)	
Dogs around the house			
No	17.4	Reference	>0.20
Yes	39.3	2.89 (1.98–4.21)	
Missing	22.8	1.44 (0.80–2.60)	
Breed			<0.20
Pure breed	18.7	Reference	
Mixed breed	37.2	2.58 (1.24–5.37)	
Missing	19.5	1.01 (0.45–2.28)	
Outdoor access			<0.20
No	9.7	Reference	
Yes	22.8	1.10 (0.48–2.52)	
Missing	10.4	0.91 (0.57–1.45)	
Hours outside per day			<0.20
Not outside	10.2	Reference	
<1 h	16.7	1.47 (0.40–5.35)	
1–5 h	22.3	2.29 (0.77–6.83)	
>5 h	35.1	4.54 (2.73–7.56)	
Missing	23.6	2.66 (1.13–6.30)	
Hunting			<0.20
No	17.3	Reference	
Yes	35.2	2.58 (1.24–5.37)	
Missing	18.8	1.15 (0.52–2.57)	
Neutered			>0.20
No	10.8	Reference	
Yes	30.5	3.72 (1.57–8.79)	
Missing	24.9	2.93 (1.40–6.14)	
Vaccinated			>0.20
No	21.1	Reference	
Yes	19.3	0.82 (0.35–1.97)	
Missing	20.4	0.95 (0.57–1.61)	
Contact with young dogs			<0.20
No	17.6	Reference	
Yes	24.8	1.63 (0.95–2.79)	
Missing	24.5	1.49 (0.93–2.37)	
Use of litter tray			<0.20
No	25.3	Reference	
Yes	16.5	0.46 (0.19–1.15)	
Missing	19.3	0.54 (0.37–0.78)	

**Table 2:** Odds-ratios (OR) with 95% confidence interval and *p*-values based on likelihood ratio tests for variables associated with *Neospora caninum* seropositivity in dogs in multivariable logistic regression analysis.

Variable	OR (95% CI)	*p-*value
Breed		<0.05
Pure breed	Reference	
Mixed breed	1.53 (0.98–2.46)	
Former stray		<0.05
No	Reference	
Yes	1.38 (0.91–2.14)	
Dogs living on cattle farms		<0.05
No	Reference	
Yes	2.30 (1.40–3.98)	
Raw meat		<0.05
No	Reference	
Yes	1.66 (1.09–2.50)	
Hunting		<0.05
No	Reference	
Yes	1.22 (0.92–1.62)	

## Discussion

Although dogs play a major role in the spread and maintenance of *N. caninum*, studies examining the occurrence of and risk factors for neosporosis in dogs are notably absent from the literature. In the present study, the prevalence of anti-*N. caninum* specific antibodies in dogs living in northeastern mainland China is reported for the first time.

To determine the seroprevalence of *N. caninum* in dogs, an IFAT was used. Serum samples with a titer of at least 1:50 were considered to be positive. Of the 476 total dog serum samples tested in this study, 95 were observed to be positive for *N. caninum* antibodies, which corresponds to an overall seropositivity rate of 20%. The prevalence recorded here is similar to the prevalence of 21.7% reported in central Poland [13]. However, the prevalence observed here was lower than that reported in northwestern Italy (36.4%) [10]. Interestingly, the seroprevalence observed here was higher than that reported in Korea (3.6%) [21], which is near northeastern mainland China.

Logistic regression analysis helped identify the risk factors associated with *N. caninum* infection. The risk factors identified were: breed, former stray dog, dogs living on cattle farms, hunting behavior, and raw meat feeding. It was observed here that seropositivity was especially high for dogs residing on cattle farms, which was possibly because of the incorrect feeding habits. In fact, dogs raised alongside livestock are exposed to tissues known to harbor *N. caninum*, including bovine fetuses and placenta, as well as other intermediate hosts including rodents and birds. Nevertheless, either with positive or negative *N. caninum*, dogs were affected by bovine tissue ingestion in the similar way [24].

In the present study, only a few dog owners fed raw meat to their dogs, resulting in borderline significance in the statistical analysis. Other previously published studies have reported an association between raw meat eating and seropositivity to *N. caninum* [14, 18]. Although few dog owners report feeding raw meat to their dogs, this behavior is still a significant risk factor. Therefore, strict regulation of raw meat feeding would effectively reduce the seroprevalence, while having little impact on dogs and their owners.

Another risk factor is hunting behavior by the dogs, through which the animals could be directly infected by ingestion of infected prey animals. This has, in fact, been demonstrated to be a source of infection and a strong risk factor. Since many dogs instinctively hunt other smaller animals, prevention of this behavior may result in a significant decrease in seroprevalence. However, mitigation of this risk factor is substantially more challenging in practice than avoiding feeding raw meat. Furthermore, 86% of dogs exhibiting hunting behavior were allowed free access to the outdoors (data not shown); the most effective mitigation strategy would be to keep dogs inside. Dog owners are not reluctant to keep them indoors. However, the multivariate analysis did not indicate that free access to the outdoors was an important predictor of infection. Therefore, actions which aim to prevent hunting behaviors while not completely restricting access to the outdoors could be effective in decreasing the risk of contracting *N. caninum* while promoting the overall health and needs of the dog. Some examples of strategies to reduce the hunting behaviors could include keeping dogs indoors at night, equipping dogs with a bib or a bell, as well as adding raw meat to the diet [19]. Though adding raw meat could reduce hunting behavior, it increases the risk of ingesting *N. caninum* cysts. Therefore, raw meat should be frozen at 2 °C prior to feeding, as this has been demonstrated to effectively kill tissue cysts [16].

The seropositivity of purebred dogs was observed to be higher than that of mixed bred dogs, which corroborates a previously published study [2]. These observations suggest that there is a genetic factor which either predisposes certain breeds to infection, or vertical transmission of the parasite may be more effective in some breeds. In contrast, other studies have reported that mixed bred dogs have higher morbidity [3, 9]. Therefore, breed-specific effects on the epidemiology of canine neosporosis remain controversial.

In addition to the above described factors, a dog being a stray at some point in its life was identified as an independent predictor for *N. caninum* infection. However, it is exceedingly difficult to establish a definitive link at the present time, and the effect was small. The potential past effect of being feral was likely due to frequent hunting. The proportion of formerly stray dogs is unlikely to represent the proportion of stray dogs in the study area. Therefore, it is expected that the prevention of domesticated dogs from straying could reduce the overall seroprevalence.

This study examines potential risk factors associated with *N. caninum* infection in dogs. Dogs were selected for the analysis because they are well established hosts, are relatively easy to collect samples from, and controlling infection in dogs would have a significant impact on the shedding of oocysts into environment. However, the amount of oocysts shed into the environment could also be affected by the population densities of dogs, the proportion of dogs defecating outside, as well as the loads of oocysts being shed by an infected dog [29]. To achieve control over the contribution of canines to environmental loads of *N. caninum*, other highly effective intervention strategies, namely reducing dog populations (e.g. by controlling the populations of stray dogs and compelling owners to spay or neuter pets prior to sexual maturity) and increasing the use of litter trays combined with appropriate filling (such as using household waste instead of toilet, organic waste, or compost) should be recommended. The patient questionnaire clearly showed that most dog owners in the northeastern region of China, where this study was conducted, used litter trays for their dogs. However, less than 26% actually used the tray frequently (data not shown).

Significant gender and vaccine differences were not observed in this study. Many studies have reported that gender imposed no effect on the emergence of *N. caninum,* and that dogs at any age could be infected [3, 4, 10]. An inverse relationship between vaccination and the number of oocysts shed exists. Although the vaccine does not induce sterilizing immunity, this control strategy may reduce the environmental parasite loads enough to prevent the spread of *N. caninum*. Unfortunately, the medical histories collected in the present study indicated that dog owners in rural areas of northeastern and mainland China did not attach importance to vaccination of their pets against this parasite. Although several of the dog owners surveyed here did in fact obtain immunizations for their dogs, the neosporosis vaccine was often not one of them.

For the current study, participants were recruited from volunteering owners. This selection method may have resulted in a population sample that is not entirely representative of the total dog population. This can have various effects. Firstly, sampling only through willing participant owners may have limited the number of dogs included in our study. Therefore, the overall prevalence may have been underestimated. Secondly, regional differences in *N. caninum* infection in dogs in the northeastern mainland may have influenced the accuracy of the risk assessment. In future studies, a broader geographical range, more active sample collection, and a larger sample size should be included in the analysis to obtain the most representative population and data possible.

## Conclusions

In the present study, breed, former stray lifestyle, residing on cattle farms, hunting behavior, and consumption of raw meat were determined to be significant risk factors for contraction of neosporosis. These risk factors can be used to develop intervention measures to limit future morbidities. As was suspected, dogs living on cattle farms presented with the highest rates of seropositivity. Therefore, it is necessary to focus more on management of dogs cohabitating and interacting with livestock. The ultimate aim is to restrict the spread of neosporosis in both dogs and cattle alike.
